# Natural Hydrazone Derivatives: Their Sources, Structures, and Bioactivities

**DOI:** 10.2174/0113895575390008250520114953

**Published:** 2025-06-04

**Authors:** Hagar M. Mohamed, Hazem G.A. Hussein, Gamal A. Mohamed, Shaimaa G.A. Mohamed, Sabrin R.M. Ibrahim

**Affiliations:** 1 Department of Medical Laboratory Analysis, College of Medical & Health Sciences, Liwa University, Abu Dhabi 41009, United Arab of Emirates;; 2 Department of Applied Medical Chemistry, Medical Research Institute, Alexandria University, Alexandria, Egypt;; 3 General Medicine Practice Program, Batterjee Medical College, Jeddah, 21442, Saudi Arabia;; 4 Department of Natural Products and Alternative Medicine, Faculty of Pharmacy, King Abdulaziz University, Jeddah, 21589, Saudi Arabia;; 5 Department of Fixed Prosthodontics, Faculty of Dentistry, The British University in Egypt, El Sherouk City, Suez Desert Road, Cairo, 11837, Egypt;; 6 Department of Chemistry, Preparatory Year Program, Batterjee Medical College, Jeddah, 21442, Saudi Arabia;; 7 Department of Pharmacognosy, Faculty of Pharmacy, Assiut University, Assiut, 71526, Egypt

**Keywords:** Hydrazone derivatives, ion sensing, bioactivities, drug discovery, nitrogen boost, electrophilic

## Abstract

Hydrazone-containing compounds are a diverse group of bioactive compounds known for their unique chemical features and diverse biological activities. Natural hydrazone derivatives have been identified from various natural sources, including bacteria, plants, fungi, and marine organisms. This work provides a comprehensive review of published works on natural hydrazone derivatives, including their sources, structural features, and biological activity in the period from 1967 to March 2025. In this work, 72 compounds were reviewed, along with 75 references being cited. The reported findings in this work highlight the therapeutic potential of these compounds in pharmaceutical research and drug discovery.

## 1 INTRODUCTION

Many natural compounds with N-N bonds, such as pyridazine, diazo, hydrazide, azoxy, hydrazine, and hydrazone, with diverse structural variations and biological activities, have been reported from different sources, particularly from actinomycetes [1, 2].

**Fig. (1):**
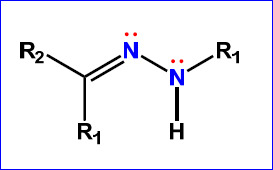
Hydrazone functional group.

Hydrazones are compounds with an R1R2C=N–NR3R4 structural formula (Fig. **1**). They are related to aldehydes and ketones and are created by replacing the oxygen of carbonyl-containing compounds with an NNH2 group [3].

Its multifaceted and versatile C=N–NH functional group includes an imine carbon with both electrophilic and nucleophilic properties, nucleophilic imine and amino-type nitrogen, an acidic N–H proton, and configurational isomerism resulting from the C=N double bond intrinsic nature [4]. The C=N double bond is stable against hydrolysis under neutral conditions due to the mesomeric effect. Also, the amino-type nitrogen boosts the coordination capacity of this group, and the acidic N–H proton contributes to anion sensing, intramolecular H-bonding, and metal coordination [5].

The hydrazone functional group can exist in Z- or E forms in the solution that both light and chemicals can initiate [6]. A study by Romero *et al.* revealed the stability of the Z form of hydrazone derivatives was due to the intramolecular hydrogen bond formation [6]. Additionally, various reports stated the hydrolytic stability of hydrazone-containing compounds under different chemical and biological conditions [7, 8]. These structural features are attributed to the hydrazone group's physical and chemical characteristics, playing a crucial role in its wide applications, such as dynamic covalent chemistry, ion sensing, and metal chelation [4]. Also, they are popular reagents in many chemical synthetic reactions, including alkenyl iodide, pyrazole ring, chiral amine, 1,3-thiazolidine-4-ones, and coumarins synthesis [2, 4].

Hydrazone-containing compounds have garnered considerable interest from organic chemists due to their chemical characteristics and diverse bioactivities, including antitubercular, antimicrobial, herbicidal, anti-inflammatory, anti-HIV, analgesic, antihypertensive, insecticidal, anticonvulsant, anticancer, cardioprotective, antiplatelet, anti-trypanosomal, antimalarial, antidepressant, anthelmintic, antiviral, antimalarial, and anti-schistosomiasis effects [9, 10]. It was found that the biological activity of several natural metabolites is enhanced by combining them with hydrazone units, which gives more options for developing novel pharmaceuticals [11-14].

Despite the popularity and richness of synthetic hydrazone derivatives, there are a limited number of naturally occurring hydrazone derivatives in comparison to synthesized hydrazones [10, 15]. These natural derivatives are commonly reported from actinomycetes, fungi, marine organisms, and plants [1]. Various published reviews have focused on N–N bond-containing compounds, in particular the synthetic ones [2, 16, 17]. Due to the lack of review addressing natural hydrazone derivatives, this work aimed to provide an in-depth review of the natural derivatives, including their structures, sources, and biological activities. Highlighting metabolites can provide an overview of their application in drug discovery and development, covering the reported data in the period from 1967 to March 2025.

## 2 SEARCH METHODOLOGY

A thorough search of scientific databases, including Scopus and Web of Science, as well as ScienceDirect and Google Scholar, was performed using keywords: “Hydrazone + Natural Products,” “Hydrazones + Biological activity,” “Hydrazones + NMR,” “Hydrazones + Isolation,” and “Natural hydrazones”. The published studies in peer-reviewed journals that focused on the structural elucidation and isolation of natural hydrazone derivatives, together with testing their bioactivity and/or mechanisms of action in the period from 1967 to March 2025were included. The studies that focused only on synthetic hydrazone molecules were excluded. Articles from non-reviewed and irrelevant journals were excluded. Further, the information from non-English articles was concluded from their English abstracts.

## 3 NATURAL HYDRAZONE DERIVATIVES AND THEIR BIOLOGICAL ACTIVITIES

In this work, seventy two natural hydrazone derivatives were discussed, along with their bioactivities. These compounds were classified according to their chemical nature into aliphatic, aromatic, and cyclic hydrazone derivatives, as shown in Fig. (**2**).

These compounds were isolated from bacteria, plants, fungi, and marine organisms (sponges, algae, and dinoflagellates). The majority of them were reported from fungi (41 compounds) and bacteria (30 compounds) as shown in Fig. (**3**). While fewer hydrazone compounds were reported, sponges (4 compounds), algae (1 compound), dinoflagellates (1 compound), and plants (1 compound). This implies that the most promising sources of hydrazone-based chemicals are bacteria and fungi, possibly due to their widespread synthesis of secondary metabolites.

These hydrazone derivatives feature distinct electrical characteristics that were reported to influence reactivity, stability, and bioactivities. The presence of conjugated −C = N-bond and functional nitrogen electron pairs gives hydrazones both electrophilic and nucleophilic properties that improve their capacity to interact with biological targets, including receptors and enzymes [18-20]. These interactions strengthen the hydrazone's ability to form H-bonds, while stereoisomerism affects bioactivity and structural diversity [18-22].

### 3.1 Aliphatic Hydrazone Derivatives

**Fig. (4):**
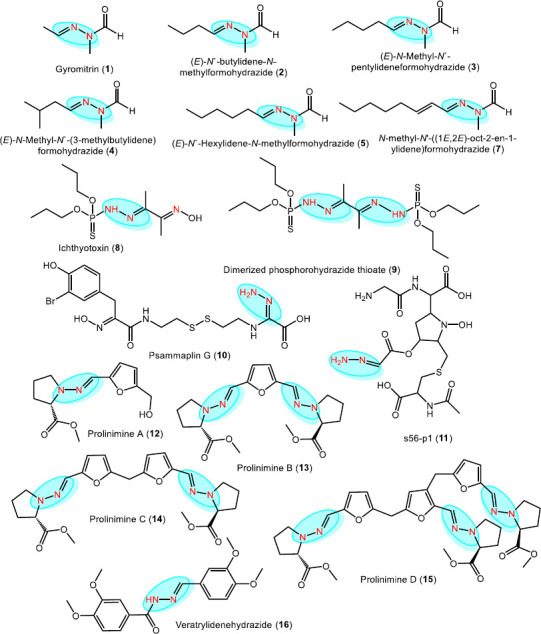
Chemical structures of aliphatic hydrazone derivatives (1–16).

Gyromitrin (1) is a water-soluble, volatile, toxic hydrazine analog that was produced by Gyromitra esculenta (false morels) and separated from this fungus by boiling. In 1975, Pyysalo separated 1, 3, 4, and 5 from the fresh false morels that were assigned by spectral analyses. Compounds 2, 3, and 5 (dose 350 mg/kg b.wt.) led to rabbit death (LD50s 100–300 mg/kg b.wt.) within 24 hr [23]. Various studies estimated the LD50s of 1 that were found to differ significantly amongst humans, rabbits, and mice (LD50s 30–50, 50–70, and 344 mg/kg, respectively). The toxicity of 1 is mostly caused by monomethyl-hydrazine that had LD50s 1.6–4.8 and 4.8–8.0 mg/kg for children and adults, respectively [24-26]. In the stomach, 1 degrades to produce poisonous hydrazines such as N-methyl-N-formylhydrazine and N-methylhydrazine that significantly depleted pyridoxine in the central nervous system (CNS), leading to a decrease in GABA (Gamma-aminobutyric acid) production. They also caused liver damage and glutathione depletion in erythrocytes [27]. A recent descriptive case study of ingestion of gyromitrin-containing mushrooms revealed that gastrointestinal symptoms were the most common clinical findings linked to their ingestions, while neurological symptoms and hepatotoxicity were less common [28]. In 1982, the red tide dinoflagellate Gymnodinium breve was found to yield compound 8, a phosphorothione hydrazone analog with phosphorus and thiol moieties [29] that was found to be toxic to rodents [30] (Table **1**). Abraham *et al.* identified an uncommon phosphorohydrazide thioate (9), a dimerized form of 8 from Lignincola laevis marine fungus [31]. This compound exhibited cytotoxic potential (IC_50_ 0.25 μM) versus the L1210 cell line (Fig. **4**) [31].

Pseudoceratina purpurea biosynthesized a novel bisulfide bromotyrosine derivative, 10, that was separated by SiO2 CC/Sephadex LH-20/HPLC and elucidated by spectral/chemical methods [30]. Compound 10 (IC_50_s 12.8 and 18.0 nM, respectively) exhibited potent DNMT (DNA methyltransferase) and HDAC (histone deacetylase) inhibitory capacity, in comparison to COP 1 (copolymer 1, IC_50_ 160 nM) in the scintillation assay [30]. These findings suggested 10 as a lead structure for developing anticancer drugs.

Heterologous expression of Streptomyces sp. SoC-090715LN-17`s amino group carrier protein (AmCP) gene cluster resulted in the identification of 11, an unusual natural dipeptide with a unique hydrazone moiety that was assumed to originate from (2S,6R)-diamino-(5R,7)-dihydroxy-heptanoic acid [32]. Mohamed *et al.* reported that Trichoderma sp. CMB-F563, derived from the gastrointestinal tract of fish, produced 12–15 that feature a unique hydrazine moiety enclosed in a Schiff base, as well as N-amino-Pro moiety. They were assigned based on spectroscopic and Marfey’s analyses, as well as biomimetic total synthesis, biosynthesis, and chemical transformations. Notably, 13 and 15 resulted from 12 by dimerization and trimerization, respectively, during isolation [33]. Compound 16 was isolated from the Thai plant Wedelia biflora, which is used in traditional medicine to alleviate fever and headaches [34].

### 3.2 Aromatic Hydrazone Derivatives

**Fig. (5):**
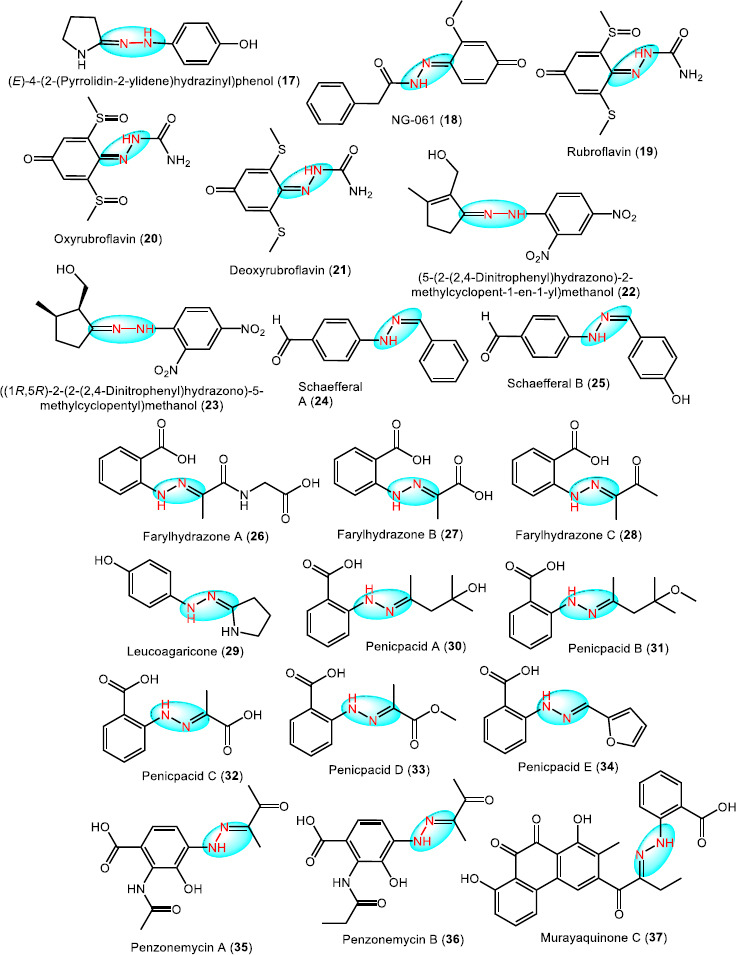
sabrin.ibrahim@bmc.edu.sa Chemical structures of natural aromatic hydrazone derivatives (17–37).

Ito *et al.* reported the separation of phenylacetic acid hydrazide analog: 18 from Penicillium minioluteum F-4627 culture broth EtOAc extract (Fig. **5**) [35].

Compound 18 exhibited a neurotrophic effect and caused neurite outgrowth in PC12 cells (Conc. 1–10 µg/mL) in the MTT assay [35]. This compound boosted and imitated the neurotrophic impact of NGF (nerve growth factor) on neurite outgrowth in PC12 cells. Whilst it demonstrated (Conc. 1 mg/mL) no antimicrobial efficacy versus yeast, bacteria, and fungi using the paper disc method. NGF is a polypeptide, neurotrophic factor, which was first isolated from the mouse submaxillary gland. It is necessary for the development and growth of neurons in both the peripheral and central nervous systems [35]. Takaishi and Fugmann reported the isolation of 19, an orange pigment from Calvatia rubroflava fruiting bodies and Calvatia craniformis, respectively [36, 37]. Further, 20 and 21, novel orange-red and red pigments were separated that were established by spectral/ECD/synthesis/quantum mechanical calculation methods. These compounds feature a semi-carbazone 1,4-benzoquinone moiety [36, 37].

Compounds 22 and 23, 2,4- dinitrophenylhydrazones were separated by Chomcheon *et al.* from Leea rubra-accompanied Dothideomycete sp. LRUB20 using SiO2 CC/Sephadex LH-20 and crystallization from MeOH and assigned using spectral/X-ray crystal analyses. Compound 23 demonstrated weak antimycobacterial potential versus Mycobacterium tuberculosis H37Ra (MIC 200 µg/mL), compared to kanamycin sulfate and isoniazid (MICs 2.0-5.0 and 0.040-0.090 µg/mL, respectively) in the microplate Alamar Blue assay (MABA). In addition, it had mild cytotoxic capacity against the Vero cell line (IC_50_ 21.7 µg/mL) [43].

Phenyl 4-hydrazinobenzaldehyde derivatives 24 and 25 were found in MeOH extracts of Agaricus silvicola and A. arvensis mushrooms (Conc. 10 mg/kg fresh weight [44]. They were identified as the chromogens that caused the caps of some Agaricus species to turn orange to red when treated with nitric acid and aniline. Kileci-Ksoll *et al.* established their structures through complete synthesis, starting with 4-aminobenzylamine [44]. Ma *et al.* identified new phenyl hydrazones, 26 and 27, from Isaria farinose using spectral/X-ray analyses. Compound 27 is like 26, except for the absence of the phenylhydrazone-linked glycine residue in 26. Unfortunately, these compounds had no notable antimicrobial or cytotoxic properties [45].

Besides, Zhang *et al.* also separated new phenylhydrazone analog, 28, along with 27 from Antarctic-derived Penicillium sp. HDN14-431 using SiO2/RP-18/Sephadex LH-20 CC/HPLC that were elucidated by spectral method/ECD calculations [46]. Compound 28 was closely similar to 27, except C-11 OH in 27 was replaced by the CH3 group in compound 28. Compounds 27 and 28 (IC_50_ > 10 μM) had no cytotoxic properties against HCT-116, K562, Hela, and A-549 cells, whereas 28 (MIC 22.5 μM) inhibited the growth of Proteusbacillus vulgaris, compared to chloramphenicol (MIC 3.13 μM) [46]. New phenylhydrazone derivatives 35 and 36 were separated from sediment-derived Streptomyces sp. SCSIO 40020 was obtained from the Pearl River Estuary using SiO2/RP-18 CC/HPLC and assigned by spectral/X-ray analyses. These compounds possess rare butanone hydrazone moiety, whereas 36 features a propionamide unit instead of the acetylamino moiety in 35. Compound 35 exhibited weak cytotoxic potential against SF-268, A549, and HepG-2 cell lines (IC_50_s 37.76, 63.19, and 31.31 µM, respectively), compared to cisplatin (IC_50_ 3.26, 1.56, and 2.42 µM, respectively) in the SRB method [47]. Chemical investigation of extract of Streptomyces sp. NA4286 derived from the gut of Forficula auricularia insect led to the identification of a new hydrazone, 37, containing phenanthrene-9,10-dione unit with a C-12 hydrazinyl benzoic acid (Fig. **5**). This compound had no activity (Conc. ˃100 μM) versus PANC-1, NCI-H23, T24, 5637, U251, and U87MG cell lines in the MTT method [48].

**Fig. (6):**
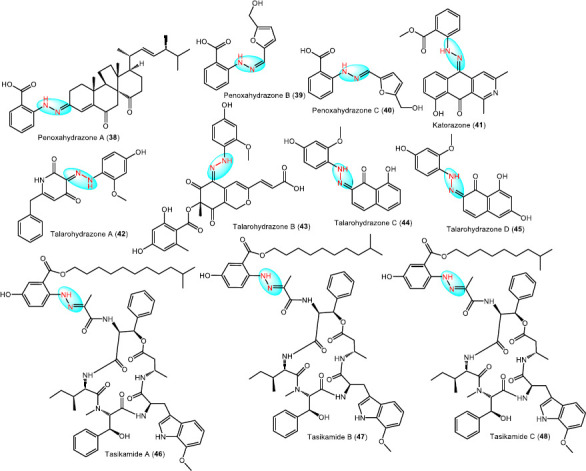
Chemical structures of natural aromatic hydrazone derivatives (38–48).

Compounds 38–40, new phenylhydrazones were separated from Penicillium oxalicum derived from deep-sea cold seep by SiO2/RP-18 CC/preparative HPLC and assigned by spectral/X-ray/ECD analyses. Compounds 39 and 40 are rare phenylhydrazone tautomers that differ in the configuration of C-10 and N-9. Compound 38 is phenylhydrazone-bearing steroid nucleus, possessing 8R/9R/10R/13R/17R/20R/24R configuration (Fig. **6**) [49].

These compounds were assessed for their inhibition capacities versus Prorocentrum donghaiense, Chattonella marina, and Heterosigma akashiwo microalgae. Compound 38 demonstrated potent inhibition of these microalgae (IC_50_s 0.68, 1.2, and 3.7 µg/mL, respectively) comparable to K2Cr2O7 (IC_50_s 1.2, 0.60, and 2.4 µg/mL, respectively), whereas 39/40 were mildly active versus C. marina and P. donghaiense (IC_50_s 17.0, and 5.4 µg/mL, respectively). They also showed mild to weak activity against marine-isolated pathogenic bacteria: Vibrio harveyi, Vibrio anguillarum, Vibrio splendidus, and Vibrio parahaemolyticus in the disk diffusion method [49].

In 2012, Abdelfattah *et al.* identified a new phenylhydrazone-2-azaquinone analog 41 from soil-associated Streptomyces sp. IFM-11299 was collected at Katori city/Chiba prefecture/Japan using flash CC/Sephadex LH-20/prep TLC (Table **2**). This compound possesses 2-azaanthraquinone and hydrazinyl benzoic acid moiety [50]. It was proposed to be biosynthesized from the condensation of methyl 2-hydrazinylbenzoate with the C-10 carbonyl group of utahmycin A that may be derived from anthranilic acid. Its bioactivity was assessed on TRAIL-resistance in AGS (TRAIL-resistant human gastric adenocarcinoma) cells by comparing cell viability in the presence and absence of TRAIL (100 ng/mL). It was noted that cell viability was 100% at 40 μM of 41. However, viability dropped to 80% of control levels upon treatment with 40 μM of 41 and 100 ng/mL TRAIL, suggesting 41 exhibited a synergistic effect when combined with TRAIL against AGS cells [50].

Talarohydrazones A-D (**42–44**) are rare phenylhydrazone alkaloids that were isolated from the deep-sea cold seep *Talaromyces amestolkiae* HDN21-0307 using OSMAC (one strain-many compound) approach, MS2LDA unsupervised substructure annotation method, and MS/MS-based molecular networking (MN) in conjunction with network annotation propagation (NAP) that were assigned by quantum chemical computations/X-ray diffraction/spectroscopic analyses [54]. Compound 42 features a unique skeleton that combines phenylhydrazone and 2,4-pyridine dione, while 43 was the first naturally occurring azadophilone, having phenylhydrazone. The cytotoxicity and antibacterial analyses revealed that 42 had weak antibacterial against S. aureus (MIC 32 μg/mL) and cytotoxic activity against NCI-H446 cells (IC_50_ 4.1 μM), compared to ciprofloxacin (MIC 16.0 µg/mL) and adriamycin (IC_50_ 0.6 µM), respectively [54]. According to Ma et al., Streptomyces tasikensis produced tasikamides A−C (46−48), a new cyclic pentapeptide with a hydrazone moiety connecting the cyclic peptide skeleton to an alkyl 5-hydroxylanthranilate moiety [55]. These metabolites are formed through two pathways. The cyclic pentapeptide is biosynthesized by the non-ribosomal peptide synthetase pathway, while the alkyl 5-hydroxylanthranilate moiety is produced and diazotized via the second pathway [54].

### 3.3 Cyclic Hydrazone Derivatives

**Fig. (7):**
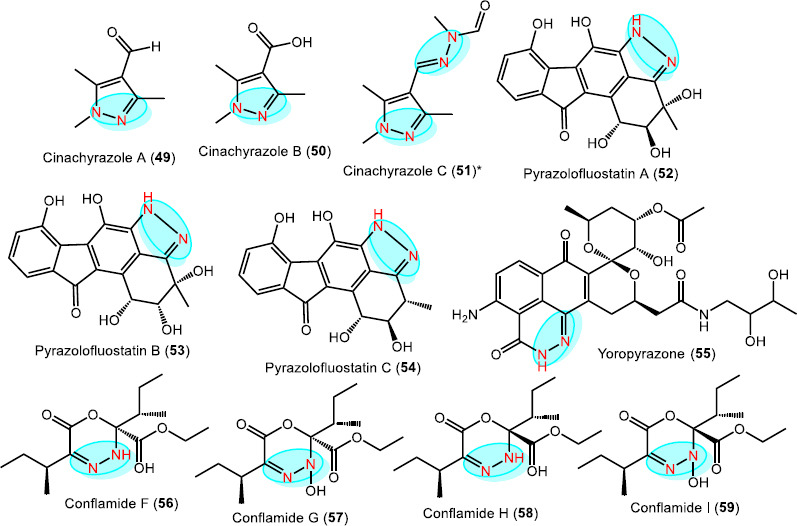
Chemical structures of natural cyclic hydrazone derivatives (49–59). * Cinachyrazole C (51) features both aliphatic and cyclic hydrazone functional groups.

Mokhlesi *et al.* separated and identified 49–51, new metabolites from Cinachyrella sp. obtained from Ambon Island/Indonesia using SiO2/Sephadex LH-20 CC/HPLC and spectral techniques, respectively (Fig. **7**). All compounds have trimethylpyrazole with an additional N’-formyl-N’-methyl-hydrazone moiety at C-4 in 51. Unfortunately, they were inactive against the L5178Y cell line (IC_50_ > 10 µM), compared to kahalalide F (IC_50_ 4.3 µM) in the MTT assay [56].

Pyrazolofluostatins A-C (52–54), new benzo[a]-fluorenes with an unparalleled carbon skeleton were separated from Micromonospora rosaria SCSIO-N160 obtained from the South China utilizing SiO2/Sephadex LH-20 CC/HPLC and elucidated by Xray/spectroscopic analyses [57]. Compounds 52–54 have a pyrazole-fused 6/5/6/6/5 pentacyclic structure with a benzo[cd]indeno [2,1-f]indazol skeleton. It is noteworthy that in the DPPH assay, 52 exhibited moderate antioxidation action (EC50 48.6 μM) in comparison to vitamin C (EC50 19.8 μM). Additionally, these metabolites showed mild antimicrobial action against B. thuringensis SCSIO-BT01, E. coli ATCC-25922, S. aureus ATCC-29213, C. albicans ATCC-10231, and B. subtilis SCSIO-BS01, as well as no cytotoxic effects on SF-268, MCF-7, NCI-H460, or HepG2 in the broth microdilution and SRB assays, respectively [57]. A study by Abdelfattah *et al.* on Streptomyces sp. IFM-11307 revealed the separation of 55 using SiO2CC/Sephadex LH-20/preparative HPLC. Compound 55 was identified by spectral analyses as a new naphthopyridazone analog, which has pyranonaphthoquinone, aminobutane-2,3-diol, and pyridazine-3(2H)-one substructure. This compound (IC_50_ 12.5 μM) demonstrated cytotoxic effectiveness against AGS cells. It also decreased AGS cell viability to 68% at 10 μM of control level. However, its combined treatment with 100 ng/mL TRAIL further lowered cell viability to 36%, revealing that naphthopyridazones could be utilized in conjunction with TRAIL to treat human gastric adenocarcinoma [58].

Conflamides F−I (56-59) are unusual heterocyclic derivatives with α-hydrazone-acid ketal moiety with numerous chiral centers that were isolated from Albatrellus confluens mushroom [59]. Their structures were ascertained by NMR/total synthesis/ECD spectra. These compounds possessed weak inhibition of Con-A (concanavalin A)- and LPS (lipopolysaccharide)-induced proliferation of T cell and B cell, respectively [59].

Luzopeptins A–C (60–62) were identified from Actinomadura luzonensis culture broth. They are cyclic deca-depsipeptides, having 3-hydroxy-6-methoxyquinaldic acid [60-63]. Their configurations were specified by synthesis/X-ray/chemical degradation [64, 65]. Compound 60 is a mono-acetyl and diacetyl analog of 61 and 62, respectively. Luzopeptins and related decadepsipeptides contain unusual acyl-substituted tetrahydropyridazine-3-carboxylic acid (Thp) moieties [64, 65].

Shi *et al.* proposed the biosynthesis of compound 60 using in-vitro biochemical and in-vivo genetic methods [66]. It was found that a multifunctional cytochrome P450 enzyme generates the hydrazone-linked 4-OH-Thp residues through catalyzing sequential oxidation steps, including a rare nitrogen-carbon bond desaturation. Furthermore, a membrane-connected acyltransferase mediates the following extracellular O-acetylation [66]. These compounds displayed marked antibacterial potential versus different Gram-positive bacteria (MICs 0.1–6.3 μg/mL), whereas 60 was the powerful (MIC 0.1–0.4 μg/mL) compound, compared to echinomycin (MIC 0.0031–0.2 μg/mL) (Table **3**) [62]. Compound 60 exhibited a significant activity versus sarcoma 180, leukemia P388, leukemia L1210, melanoma B16, and LEWIS lung carcinoma in mice. It was found that 60 was 100 folds more active than mitomycin C versus all cell lines except for sarcoma 180, which was 300 times more sensitive to 60 than mitomycin C [62].

**Fig. (8):**
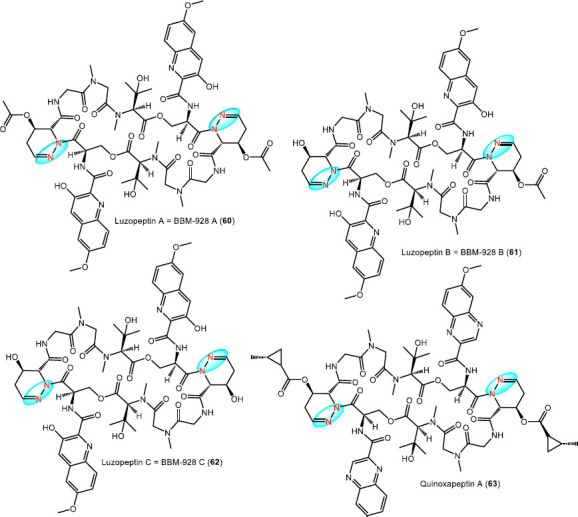
Chemical structures of natural cyclic hydrazone derivatives (60–63).

Compound 60 is an actinoleukin-like antibiotic with potential anticancer properties, as it links with DNA. This compound strongly binds to DNA, engaging 11 nucleotides per binding site [70, 71] (Fig. **8**). This interaction happens in two primary ways: type I (intramolecular cross-linking), where 60 binds to distinct areas on the same DNA molecule, or type II (intermolecular cross-linking), where it attaches to two separate DNA molecules. It was assumed that type I interaction is the primary mechanism of 60 actions [71, 72]. Furthermore, 62 is linked with DNA similarly to 60 and 61. However, the differences in their ability to cross the cell membrane could be responsible for the variations in their anticancer and cytotoxicity actions [72].

Another study by Rose *et al.* showed that 60 was highly effective against P388 and B16 murine tumor models. Besides, it had high DNA binding and apparently lacked hepatotoxicity, myelotoxicity, or nephrotoxicity [73]. Additionally, 60–62 showed the ability to prohibit (% RT`s inhibition 89.0-100.0, Conc. 10 μg/mL) double and single mutations that are the cause of the developing resistance to reverse transcriptase inhibitors. It is noteworthy that 62 at noncytotoxic levels hindered HIV replication in infected MT-4 cells [74], whereas 60 (IC_50_s 7.0 and 68.0 nM, respectively) suppressed HIV-1 RT and HIV-2 RT [68].

**Fig. (9):**
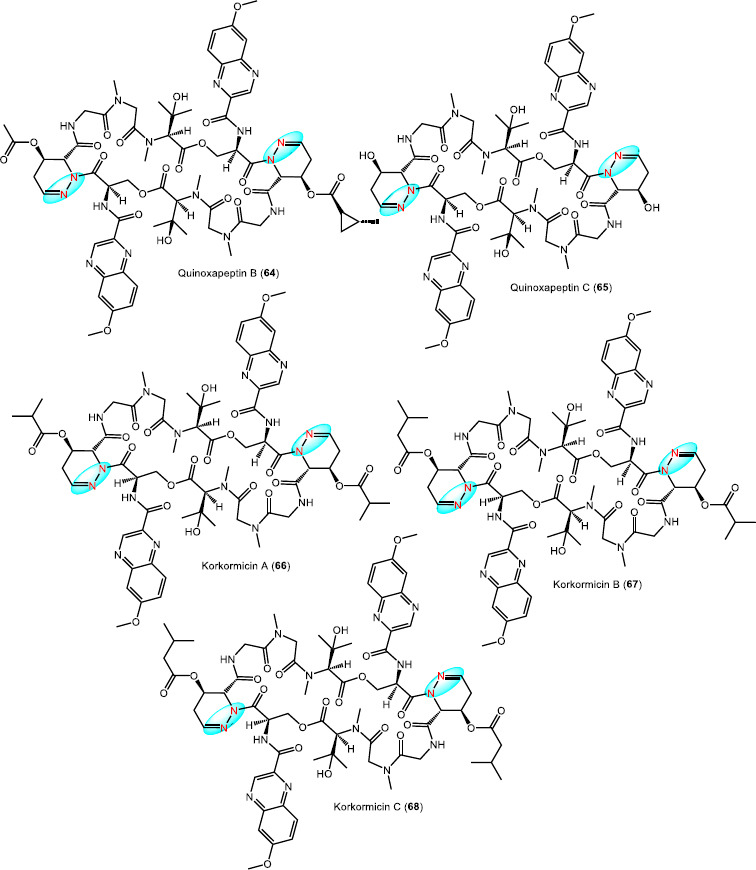
Chemical structures of natural cyclic hydrazone derivatives (64–68).

Compounds 60–62 (% RT inhibition 89.0, 97.0, and 100.0, respectively, Conc. 10 μg/mL) showed the ability to impede double and single mutants, accounting for developing clinical resistance to (reverse transcriptase) RT inhibitors. Besides, 60 (IC_50_s 7.0 and 68.0 nM, respectively) prohibited HIV-1 RT and HIV-2 RT [68], whereas 62 at noncytotoxic levels hindered HIV replication in infected MT-4 cells [68, 74]. Additionally, a study by Borger *et al.* revealed that 60–62 possessed antiviral capabilities (IC_50_s 6.0, 3.0, and 0.4 μM, respectively) in comparison to sandramycin (IC_50_ 2.0 μM). In the MTT assay, they also exhibited powerful cytotoxic activity against the HCT-116 and L1210 cell lines (IC_50_s 0.3 and 0.008 for 60 and 30.0 and 30.0 nM for 61); however, at ˃100 nM, 62 was inactive in comparison to sandramycin (IC_50_s 0.007 and 0.001 nM, respectively [65, 67]. The acute study of these compounds in mice revealed that 60 was 55 folds more toxic than mitomycin. However, it was less toxic than echinomycin. Compound 60 was 74 times more toxic than mitomycin C, whereas 61 and 62 were 1.4 and 6.2 times, respectively, less toxic than 60 (Fig. **9**) [62].

Compounds 63–65 are new chromodepsipeptides, which were separated from Norcardioform actinomycete MA7095 associated with Betula papyrifera bark obtained from Alaska's Denali National Park. These compounds feature the same peptide framework as 60 except for the acyl substituent attached to the tetrahydropyrazidine-3-carboxylic moiety [65, 67].

Compounds 63 and 64 were found to have strong selective HIV-1 and HIV-2 RT inhibitory activity. They were equally effective against a double mutant of HIV-1 RT that causes the emergence of resistance to the approved RT inhibitors, as well as two single mutations. Whilst they possessed no inhibitory efficacy against human DNA polymerases α, β, γ, and δ [68]. Additionally, 63–65 possessed notable antiviral activity against HIV-1 RT (IC_50_s 0.6, 0.9, and 0.3 μM, respectively), compared to sandramycin (IC_50_ 2.0 μM). These metabolites also exhibited potent cytotoxic properties in the MTT assay against L1210 and HCT-116 cell lines (IC_50_s 0.3/1.0 nM for 63 and 2.0/7.0 nM for 64). However, 65 was inactive in comparison to sandramycin (IC_50_s 0.001 and 0.007 nM, respectively) [65, 67] (Table **4**). It is noteworthy that quinoxapeptins were more effective at inhibiting HIV-1 reverse transcriptase, whereas luzopeptins were found to be more effective cytotoxic agents [65, 67].

**Fig. (10):**
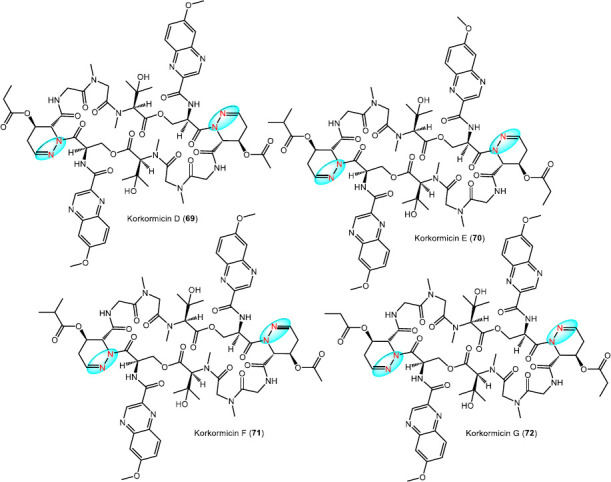
Chemical structures of natural cyclic hydrazone derivatives (69–72).

New luzopeptin structurally similar depsipeptide antitumor antibiotics: 64–72 were separated from soil-associated Micromonospora sp. C39500 by SiO2 CC/HPLC (Fig. **10**) [69]. The in-vivo anticancer efficacy of compound 66 was assessed against mice with lymphocytic leukemia P388. This compound (doses 0.05 and 0.2 mg/kg/injection) displayed the highest efficacy (life span 45% increase), whereas it was toxic at dose 0.7 mg/kg injection. Also, it was efficient (MICs 0.13-0.5 μg/mL) against E. faecalis (A20688, A25707, and A25708) and S. aureus (A9537, A20698, and A24407) Kitagaki and Yang reported that 66 can intercalate to DNA [69]. Additionally, it caused p53 phosphorylation, leading to inhibiting p53 degradation and activating p53-dependent transcription [75]. Furthermore, it predominantly promoted apoptosis in transformed cells expressing wild-type p53, suggesting 66 is a possible anticancer treatment for malignancies with wild-type p53 [75].

## 4 STUDY LIMITATIONS

Despite this work offering a thorough review of natural hydrazone derivatives, certain limitations must be acknowledged. First, compared to their synthetic derivatives, there is limited data on natural hydrazone derivatives, which limits a more comprehensive understanding of their complete structural variety and pharmacological potential. Also, there are gaps in understanding the biosynthesis, metabolism, and ecological roles of these derivatives because so many studies have concentrated on synthetic hydrazones. Second, the reported bioactivity outcomes are based on in-vitro and limited in-vivo studies, with a lack of clinical trials to support their pharmacological significance.

Furthermore, there is a lack of information on the pharmacokinetics and toxicity of these derivatives. Despite some derivatives showing cytotoxicity against cancer cells, their bioavailability, safety profiles, and possible adverse effects on normal cells remain unstudied, requiring more in-vivo research and clinical trials. These metabolites are only found especially in fungi, bacteria, and marine organisms, making their sustainable and large-scale production difficult. This could be resolved by using advanced approaches like microbial fermentation and total synthesis.

## CONCLUSION

Nitrogen-containing compounds are distinctive chemical components that have a wide range of biological effects. Among them, hydrazone derivatives have drawn particular attention from researchers due to their well-known chelating capabilities, structural flexibility, and a wide variety of medicinal applications. In this work, 72 natural hydrazone derivatives reported from different sources, including bacteria, plants, fungi, and marine organisms, were discussed.

These compounds possessed antimicrobial, cytotoxic, anti-viral, and neuroprotective properties. For example, psammaplin G, a bromotyrosine-derived hydrazone metabolite, showed significant dual inhibitory activity against DNMT and HDAC. This dual inhibition can lead to epigenetic modulation in cancer cells, reactivating tumor suppressor genes and inducing cancer cell apoptosis, suggesting Psammaplin G’s potential as a multi-target anticancer agent. Compounds 41 and 55 have shown significant activity in sensitizing gastric adenocarcinoma cells to tumor necrosis factor-related apoptosis-inducing ligand (TRAIL) therapy. This synergistic effect suggested 41 and 55’s potential to overcome TRAIL resistance in cancer cells, underscoring their relevance in combination cancer therapies. Luzopeptins A–C (60–62) revealed significant antibacterial, antiviral, and anticancer potential. Compound 60 exhibited remarkable potency against various Gram-positive bacteria and numerous cancer cell lines, demonstrating superior efficacy compared to standards like mitomycin C. Additionally, these compounds showed promise in inhibiting HIV replication, specifically targeting reverse transcriptase. The newly identified chromodepsipeptides (63–65) further displayed inhibitory activity against HIV and cytotoxic effects.

These compounds' diverse structures and biological functions provide significant opportunities for future medicinal chemistry and pharmacological research. The capacity of natural hydrazone derivatives to target several biological pathways makes them interesting candidates for therapeutic development and drug discovery. More investigation into their mechanisms of action, structure-activity relations, and possible clinical applications is crucial to fully harnessing their therapeutic benefits.

Collectively, natural hydrazone-containing metabolites represent an understudied yet significant class of biometabolites, with promising potential for treating cancer, viral diseases, and microbial infections. Future research should concentrate on defining their biological properties, enhancing their bioavailability, and assessing their therapeutic potential to fully understand their application in modern medicine.

## Figures and Tables

**Fig. (2):**
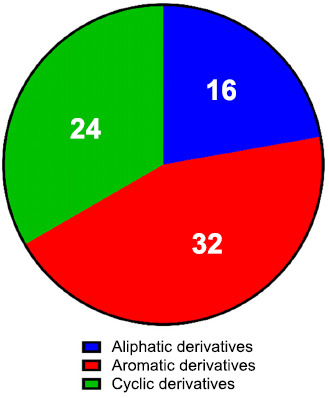
Different classes of natural hydrazone derivatives and the number of compounds.

**Fig. (3):**
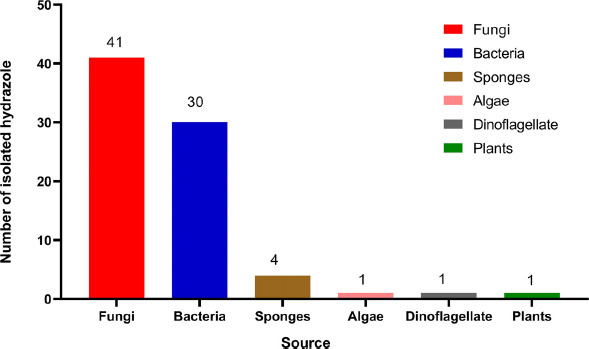
Numbers of hydrazone-containing natural products reported from different sources.

**Table 1:** List of natural aliphatic hydrazone derivatives (Molecular weight and formulae, source, and locations).

**Compound Name**	**Mol. Wt.**	**Mol. Formula**	**Source (type, family), Location**	**Refs.**
Aliphatic hydrazone derivatives	-	-	-	-
Gyromitrin = Ethylidene gyromitrin (1)	100	C_4_H_8_N_2_O	*Gyromitra esculenta *(Fungus, Discinaceae), southern Finland	[23, 38, 39]
(*E*)-*N*`-butylidene-N-methylformohydrazide (2)	128	C_7_H_12_N_2_O	*Gyromitra esculenta *(Fungus, Discinaceae)	-
(*E*)-*N*-Methyl-*N*`-pentylideneformohydrazide (3)	142	C_7_H_14_N_2_O	*Gyromitra esculenta *(Fungus, Discinaceae), southern Finland	[23]
(*E*)-*N*-Methyl-*N*`-(3-methylbutylidene)formohydrazide (4)	142	C_7_H_14_N_2_O	*Gyromitra esculenta *(Fungus, Discinaceae), southern Finland	[23]
(*E*)-*N*`-Hexylidene-*N*-methylformohydrazide (5)	156	C_8_H_16_N_2_O	*Gyromitra esculenta *(Fungus, Discinaceae), southern Finland	[23]
(*E*)-*N*-Methyl-*N*`-octylideneformohydrazide (6)	184	C_10_H_20_N_2_O	*Gyromitra esculenta *(Fungus, Discinaceae), southern Finland	[40, 41]
*N*-Methyl-*N*`-((1*E*,2*E*)-oct-2-en-1- ylidene)formohydrazide (7)	182	C_10_H_18_N_2_O	*Gyromitra esculenta *(Fungus, Discinaceae), southern Finland	[40, 41]
Ichthyotoxin = (*E*)-2-(1-Methyl-2-oxopropylidene)phosphorohydrazidothioate (*E*)-Oxime = Gb-4 (8)	295	C_10_H_22_N_3_O_3_PS	*Ptychodicus breve *(Dinoflagellate, Gymnodiniaceae), Gulf Coast of Florida	[29]
-	-	-	*Gymnodinium breve *(Dinoflagellate, (Gymnodiniaceae), Gulf coast of Florida, USA	[29]
Dimerized phosphorohydrazide thioate (9)	474	C_16_H_36_N_4_O_4_P_2_S_2_	*Lignincol laevis *(Fungus, Halosphaeriaceae), marine fungus, England	[31]
Psammaplin G (10)	493	C_15_H_20_BrN_5_O_5_S_2_	*Pseudoceratina purpurea *(Sponge, Aplisysinellidae), Papua New Guinea	[30]
s56-p1 (11)	478	C_16_H_26_N_6_O_9_S	*Streptomyceslividans* (TK23) *Streptomyces* sp. (SoC090715LN-17) (Bacteria, Streptomycetaceae), Tokyo, Japan	[32]
Prolinimine A (12)	252	C_12_H_16_N_2_O_4_	*Trichoderma* sp. (CMB-F563) (Fungus, Hypocreaceae), gastrointestinal tract of a specimen of Mugil mullet fish	[33]
-	-	-	*Evlachovaea* sp. (CMB-F563) (fungus, Cordycipitaceae), gastrointestinal tract of a specimen of Mugil mullet fish	[42]
Prolinimine B (13)	376	C_18_H_24_N_4_O_5_	*Trichoderma* sp. (CMB-F563) (Fungus, Hypocreaceae), gastrointestinal tract of a specimen of Mugil mullet fish	[33]
-	-	-	*Evlachovaea* sp. (CMB-F563) (Cordycipitaceae), gastrointestinal tract of a specimen of Mugil mullet fish	[42]
Prolinimine C (14)	456	C_23_H_28_N_4_O_6_	*Trichoderma* sp. (CMB-F563) (Fungus, Hypocreaceae), gastrointestinal tract of a specimen of Mugil mullet fish	[33]
-	-	-	*Evlachovaea* sp. (CMB-F563) (fungus, Cordycipitaceae), gastrointestinal tract of a specimen of Mugil mullet fish	[42]
Prolinimine D (15)	690	C_35_H_42_N_6_O_9_	*Trichoderma* sp. (CMB-F563) (Fungus, Hypocreaceae), gastrointestinal tract of a specimen of Mugil mullet fish	[33]
-	-	-	*Evlachovaea* sp. (CMB-F563) (fungus, Cordycipitaceae), gastrointestinal tract of a specimen of Mugil mullet fish	[42]
Veratrylidenehydrazide (16)	344	C_18_H_20_N_2_O_5_	*Wedelia bijlora *(Plant, Asteraceae), Samut Sakron, Thailand	[34]

**Table 2:** List of natural aromatic hydrazone derivatives (Molecular weight and formulae, source, and locations).

**Compound Name**	**Mol. Wt.**	**Mol. Formula**	**Source (type, family), Location**	**Refs.**
(*E*)-4-(2-(Pyrrolidin-2-ylidene)hydrazinyl)phenol (17)	191	C_10_H_13_N_3_O	*Agaricus xanthoderma* (Fungus, Agaricaceae)	[51]
NG-061 (18)	270	C_15_H_14_N_2_O_3_	*Penicillium minioluteum *(F-4627) (Fungus, Trichocomaceae), dead leaf, Niigata City in Japan	[35, 52]
Rubroflavin (19)	273	C_9_H_11_N_3_O_3_S_2_	*Calvatia craniiformis* (Fungus, Agaricaceae), Kamiyama-Chyo, Tokushima, Japan	[37, 47]
Deoxyrubroflavin (20)	257	C_9_H_11_N_3_O_3_S_2_	*Calvatia rubro-flava* (Fungus, Agaricaceae), Marshall Forest, Great Smoky Mountains, Georgia, USA	[36]
Oxyrubroflavin (21)	289	C_9_H_11_N_3_O_4_S_2_	*Calvatia rubro-flava* (Fungus, Agaricaceae), Marshall Forest, Great Smoky Mountains, Georgia, USA	[36]
(5-(2-(2,4-Dinitrophenyl)hydrazono)-2- methylcyclopent-1-en-1-yl)methanol (22)	306	C_13_H_14_N_4_O_5_	*Dothideomycete *sp. (LRUB20) (Fungus, Dothideomycetes), Thai medicinal plant, Leea rubra Blume ex Spreng. (family Leeaceae), forest areas of Pitsanulok, Thailand	[43]
((1*R*,5*R*)-2-(2-(2,4-Dinitrophenyl)hydrazono)-5-methylcyclopentyl)methanol (23)	308	C_13_H_16_N_4_O_5_	*Dothideomycete *sp. (LRUB20) (Fungus, Dothideomycetes), Thai medicinal plant, Leea rubra Blume ex Spreng. (family Leeaceae), forest areas of Pitsanulok, Thailand	[43]
Schaefferal A (24)	224	C_14_H_12_N_2_O	*Agaricus silvicola, *(Fungus, Agaricaceae), Stadtwald, Germany	[44]
Schaefferal B (25)	240	C_14_H_12_N_2_O_2_	*Agaricus silvicola *(Fungus, Agaricaceae), Stadtwald, Germany	[44]
Farylhydrazone A (26)	279	C_12_H_13_N_3_O_5_	*Isaria farinose *(Fungus, Cordycipitaceae), Linzhi, Tibet	[45]
Farylhydrazone B (27)	222	C_10_H_10_N_2_O_4_	*Isaria farinose *(Fungus, Cordycipitaceae), Linzhi, Tibet	[45]
-	-	-	*Penicillium* sp. HDN14-431 (Fungus, Trichocomaceae), soil of mesolittoral zone, the Antarctic	[46]
Farylhydrazone C (28)	220	C_11_H_12_N_2_O_3_	*Penicillium* sp. HDN14-431 (Fungus, Trichocomaceae), soil of mesolittoral zone, the Antarctic	[46]
Leucoagaricone (29)	191	C_10_H_13_N_3_O	*Agaricus xanrhodenna* (Fungus, Agaricaceae)	[51]
Penicpacid A (30)	250	C_13_H_18_N_2_O_3_	*Penicillium paneum* (SD-44) (Fungus, Trichocomaceae), sediment, South China deep sea	[53]
Penicpacid B (31)	264	C_14_H_20_N_2_O_3_	*Penicillium paneum* (SD-44) (Fungus, Trichocomaceae), sediment, South China deep sea	[53]
Penicpacid C (32)	222	C_10_H_10_N_2_O_4_	*Penicillium paneum* (SD-44) (Fungus, Trichocomaceae), sediment, South China deep sea	[53]
Penicpacid D (33)	236	C_11_H_12_N_2_O_4_	*Penicillium paneum* (SD-44) (Fungus, Trichocomaceae), sediment, South China deep sea	[53]
Penicpacid E (34)	230	C_12_H_10_N_2_O_3_	*Penicillium paneum* (SD-44) (Fungus, Trichocomaceae), sediment, South China deep sea	[53]
Penzonemycin A (36)	293	C_13_H_15_N_3_O_5_	*Streptomyces* sp. (SCSIO 40020) (Bacteria, Streptomycetaceae), marine sediment, Pearl River Estuary in China	[47]
Penzonemycin B (36)	307	C_14_H_17_N_3_O_5_	*Streptomyces* sp. (SCSIO 40020) (Bacteria, Streptomycetaceae), marine sediment, Pearl River Estuary in China	[47]
Murayaquinone C (37)	472	C_26_H_20_N_2_O_7_	*Streptomyces* sp. (NA4286) (Bacteria, Streptomycetaceae), gut of an insect, *Forficula auricularia*	[48]
Penoxahydrazone A (38)	558	C_35_H_46_N_2_O_4_	*Penicillium oxalicum *(Fungus, Trichocomaceae), sediments, deep sea cold seep	[49]
Penoxahydrazone B (39)	260	C_13_H_12_N_2_O_4_	*Penicillium oxalicum *(Fungus, Trichocomaceae), sediments, deep sea cold seep	[49]
Penoxahydrazone C (40)	260	C_13_H_12_N_2_O_4_	*Penicillium oxalicum *(Fungus, Trichocomaceae), sediments, deep sea cold seep	[49]
Katorazone (41)	401	C_23_H_19_N_3_O_4_	*Streptomyces* sp. (IFM 11299) (Bacteria, Streptomycetaceae), soil, Katori, Chiba prefecture, Japan	[50]
Talarohydrazone A (42)	351	C_19_H_17_N_3_O_4_	*Talaromyces amestolkiae* (HDN21-0307) (Fungus, Trichocomaceae), deep-sea cold seep sediment, South China Sea	[54]
Talarohydrazone B (43)	564	C_28_H_24_N_2_O_11_	*Talaromyces amestolkiae* (HDN21-0307) (Fungus, Trichocomaceae), deep-sea cold seep sediment, South China Sea	[54]
Talarohydrazone C (44)	310	C_17_H_14_N_2_O_4_	*Talaromyces amestolkiae* (HDN21-0307) (Fungus, Trichocomaceae), deep-sea cold seep sediment, South China Sea	[54]
Talarohydrazone D (45)	326	C_17_H_14_N_2_O_5_	*Talaromyces amestolkiae* (HDN21-0307) (Fungus, Trichocomaceae), deep-sea cold seep sediment, South China Sea	[54]
Tasikamide A (46)	1142	C_63_H_82_N_8_O_12_	*Streptomyces tasikensis* (P46) (Bacteria, Streptomycetaceae), Pulau Ubin quarry lake, Singapore	[55]
Tasikamide B (47)	1128	C_62_H_80_N_8_O_12_	*Streptomyces tasikensis* (P46) (Bacteria, Streptomycetaceae), Pulau Ubin quarry lake, Singapore	[55]
Tasikamide C (48)	1114	C_61_H_78_N_8_O_12_	*Streptomyces tasikensis* (P46) (Bacteria, Streptomycetaceae), Pulau Ubin quarry lake, Singapore	[55]

**Table 3:** List of natural cyclic hydrazone derivatives (Molecular weight and formulae, source, and locations).

**Compound Name**	**Mol. Wt.**	**Mol. Formula**	**Source (type, family), Location**	**Refs.**
Cinachyrazole A (49)	138	C_7_H_10_N_2_O	*Cinachyrella* sp. (Sponge, Tetillidae), Ambon, Indonesia	[56]
Cinachyrazole B (50)	154	C_7_H_10_N_2_O_2_	*Cinachyrella* sp. (Sponge, Tetillidae), Ambon, Indonesia	[56]
Cinachyrazole C (51)	194	C_9_H_12_N_4_O	*Cinachyrella* sp. (Sponge, Tetillidae), Ambon, Indonesia	[56]
Pyrazolofluostatin A (52)	354	C_18_H_14_N_2_O_6_	*Micromonospora rosaria* (SCSIO N160) (Bacteria, Micromonosporaceae), South China Sea	[57]
Pyrazolofluostatin B (53)	354	C_18_H_14_N_2_O_6_	*Micromonospora rosaria* (SCSIO N160) (Bacteria, Micromonosporaceae), South China Sea	[57]
Pyrazolofluostatin C (54)	338	C_18_H_14_N_2_O_5_	*Micromonospora rosaria* (SCSIO N160) (Bacteria, Micromonosporaceae), South China Sea	[57]
Yoropyrazone (55)	572	C_27_H_32_N_4_O_10_	*Streptomyces* sp. (IFM 11307) (Bacteria, Streptomycetaceae), soil, Katori, Chiba prefecture, Japan	[58]
Conflamide F (56)	284	C_14_H_24_N_2_O_4_	*Albatrellus confluens *(Fungus, Polyporaceae), Hutiao Gorge in southwestern China, Yunnan	[59]
Conflamide G (57)	300	C_14_H_24_N_2_O_5_	*Albatrellus confluens *(Fungus, Polyporaceae), Hutiao Gorge in southwestern China, Yunnan	[59]
Conflamide H (58)	284	C_14_H_24_N_2_O_4_	*Albatrellus confluens *(Fungus, Polyporaceae), Hutiao Gorge in southwestern China, Yunnan	[59]
Conflamide I (59)	300	C_14_H_24_N_2_O_5_	*Albatrellus confluens *(Fungus, Polyporaceae), Hutiao Gorge in southwestern China, Yunnan	[59]
Luzopeptin A = BBM-928 A (60)	1426	C_64_H_78_N_14_O_24_	*Actinomadura luzonensis* (ATCC 31491) (Bacteria, Thermomonosporaceae), Soil, Luzon Island, Philippines	[60-63, 65]
Luzopeptin B = BBM-928 B (61)	1400	C_63_H_80_N_14_O_23_	*Actinomadura luzonensis* (ATCC 31491) (Bacteria, Thermomonosporaceae), Soil, Luzon Island, Philippines	[60, 62, 65]
Luzopeptin C = BBM-928 C (62)	1342	C_60_H_74_N_14_O_22_	*Actinomadura luzonensis* (ATCC 31491) (Bacteria, Thermomonosporaceae), Soil, Luzon Island, Philippines	[60, 62, 65]
Quinoxapeptin A (63)	1476	C_68_H_84_N_16_O_22_	*Streptoverticillium album* (Q132-6) (Bacteria, Streptomycetaceae), bark disc, Betula papyrifera, Denali National Part, Alaska https://en.wikipedia.org/wiki/Thermomonosporaceae	[65, 67, 68]
Quinoxapeptin B (64)	1436	C_65_H_80_N_16_O_22_	*Streptoverticillium album* (Q132-6) (Bacteria, Streptomycetaceae), bark disc, Betula papyrifera, Denali National Part, Alaska https://en.wikipedia.org/wiki/Thermomonosporaceae	[65, 67, 68]
Quinoxapeptin C (65)	1312	C_58_H_72_N_16_O_20_	*Streptoverticillium album* (Q132-6) (Bacteria, Streptomycetaceae), bark disc, Betula papyrifera, Denali National Part, Alaska https://en.wikipedia.org/wiki/Thermomonosporaceae	[65, 67, 68]
Korkormicin A (66)	1468	C_67_H_88_N_16_O_22_	*Micromonospora* sp (C39500) (Bacteria, Micromonosporaceae), Cultured	[69]
Korkormicin B (67)	1482	C_68_H_90_N_16_O_22_	*Micromonospora* sp (C39500) (Bacteria, Micromonosporaceae), Cultured	[69]
Korkormicin C (68)	1496	C_69_H_92_N_16_O_22_	*Micromonospora* sp (C39500) (Bacteria, Micromonosporaceae), Cultured	[69]
Korkormicin D (69)	1426	C_64_H_82_N_16_O_22_	*Micromonospora* sp (C39500) (Bacteria, Micromonosporaceae), Cultured	[69]
Korkormicin E (70)	1454	C_66_H_86_N_16_O_22_	*Micromonospora* sp (C39500) (Bacteria, Micromonosporaceae), Cultured	[69]
Korkormicin F (71)	1440	C_65_H_84_N_16_O_22_	*Micromonospora* sp (C39500) (Bacteria, Micromonosporaceae), Cultured	[69]
Korkormicin G (72)	1424	C_64_H_80_N_16_O_22_	*Micromonospora* sp (C39500) (Bacteria, Micromonosporaceae), Cultured	[69]

**Table 4:** Biological activity of the most active natural hydrazone derivatives.

Compound Name	Biological Activity	Assay, Organism or Cell Line	Biological Results		Refs.
			Compound	Positive Control	
Psammaplin G (10)	DNMT inhibition	Scintillation/^3^H]acetate release	12.8 nM (IC_50_)	COP -1 160.0 nM (IC_50_)	[30]
-	HDAC inhibition	Scintillation/^3^H]acetate release	18.0 nM (IC_50_)	-	-
Farylhydrazone C (28)	Antibacterial	Broth microdilution/ *Proteusbacillus vulgaris*	22.5 μM (MIC)	Chloramphenicol 3.13 μM (MIC)	[46]
Talarohydrazone A (42)	Antibacterial	Broth microdilution/ *Staphylococcus aureus*	32.0 μg/mL (MIC)	Ciprofloxacin 16.0 μg/mL (MIC)	[54]
Pyrazolofluostatin A (52)	Antioxidant	DPP-H	48.6 μM (EC50)	Vitamin C 19.8 μM (EC50)	[57]
Luzopeptin A = BBM-928 A (60)	Antimicrobial	Serial agar diffusion/ *S. aureus* FDA 209 P	0.2 μg/mL (MIC)	Echinomycin 0.0125 μg/mL (MIC)	[62]
	-	Serial agar diffusion/ *S. aureus* Smith	0.2 μg/mL (MIC)	Echinomycin 0.0125 μg/mL (MIC)	[62]
	-	Serial agar diffusion/*S. pyogenes* S-23	0.1 μg/mL (MIC)	Echinomycin 0.0063 μg/mL (MIC)	[62]
-	-	Serial agar diffusion/ *Sarcina lutea* PCI 1001	0.2 μg/mL (MIC)	Echinomycin 0.0063 μg/mL (MIC)	[62]
-	-	Serial agar diffusion/ *Micrococcus flavus* D 12	0.2 μg/mL (MIC)	Echinomycin 0.0063 μg/mL (MIC)	[62]
-	-	Serial agar diffusion/ *Corynebacterium Xerosis* 53 K-1	0.4 μg/mL (MIC)	Echinomycin 0.2 μg/mL (MIC)	[62]
-	-	Serial agar diffusion/*B. subtilis* PCI 219	0.4 μg/mL (MIC)	Echinomycin 0.0031 μg/mL (MIC)	[62]
-	-	Serial agar diffusion/*B. megaterium* D2	0.2 μg/mL (MIC)	Echinomycin 0.1 μg/mL (MIC)	[62]
-	-	Serial agar diffusion/*B. anthracis* A 9504	0.2 μg/mL (MIC)	Echinomycin 0.025 μg/mL (MIC)	[62]
-	-	Serial agar diffusion/*Mycobacterium smegmatis* 607 D 87	0.4 μg/mL (MIC	Echinomycin 6.3 μg/mL (MIC)	[62]
-	-	Serial agar diffusion/ *Mycobacterium phlei* D 88	0.4 μg/mL (MIC	Echinomycin 6.2 μg/mL (MIC)	[62]
-	Antiviral	HIV-1	6.0 μM (IC_50_)	Sandramycin 2.0 μM (IC_50_)	[65, 67]
-	-	HIV-1	7.0 nM (IC_50_)	-	[68]
-	-	HIV-2	68.0 nM (IC_50_)	-	[68]
-	Cytotoxicity	MTT/L1210	0.008 nM (IC_50_)	Sandramycin 0.001 nM (IC_50_)	[66]
-	-	MTT/HCT-116	0.3 nM (IC_50_)	Sandramycin 0.007 nM (IC_50_)	[66]
Luzopeptin B = BBM-928 B (61)	Antimicrobial	Serial agar diffusion/ *S. aureus* FDA 209 P	0.8 μg/mL (MIC)	Echinomycin 0.0125 μg/mL (MIC)	[62]
	-	Serial agar diffusion/ *S. aureus* Smith	0.4 μg/mL (MIC)	Echinomycin 0.0125 μg/mL (MIC)	[62]
	-	Serial agar diffusion/*S. pyogenes* S-23	0.2 μg/mL (MIC)	Echinomycin 0.0063 μg/mL (MIC)	[62]
	-	Serial agar diffusion/ *Sarcina lutea* PCI 1001	0.2 μg/mL (MIC)	Echinomycin 0.0063 μg/mL (MIC)	[62]
-	-	Serial agar diffusion/ *Micrococcus flavus* D 12	0.8 μg/mL (MIC)	Echinomycin 0.0063 μg/mL (MIC)	[62]
-	-	Serial agar diffusion/ *Corynebacterium Xerosis* 53 K-1	1.6 μg/mL (MIC)	Echinomycin 0.2 μg/mL (MIC)	[62]
-	-	Serial agar diffusion/*B. subtilis* PCI 219	1.6 μg/mL (MIC)	Echinomycin 0.0031 μg/mL (MIC)	[62]
-	-	Serial agar diffusion/*B. megaterium* D2	0.4 μg/mL (MIC)	Echinomycin 0.1 μg/mL (MIC)	[62]
-	-	Serial agar diffusion/*B. anthracis* A 9504	0.2 μg/mL (MIC)	Echinomycin 0.025 μg/mL (MIC)	[62]
-	-	Serial agar diffusion/*Mycobacterium smegmatis* 607 D 87	0.4 μg/mL (MIC)	Echinomycin 6.3 μg/mL (MIC)	[62]
-	-	Serial agar diffusion/ *Mycobacterium phlei* D 88	0.4 μg/mL (MIC)	Echinomycin 6.2 μg/mL (MIC)	[62]
-	Antiviral	HIV-1	3.0 μM (IC_50_)	Sandramycin 2.0 μM (IC_50_)	[65]
-	Cytotoxicity	MTT/L1210	30.0 nM (IC_50_)	Sandramycin 0.001 nM (IC_50_)	[65]
-	-	MTT/HCT-116	30.0 nM (IC_50_)	Sandramycin 0.007 nM (IC_50_)	[65]
Luzopeptin C = BBM-928 C (62)	Antimicrobial	Serial agar diffusion/ *S. aureus* FDA 209 P	6.3 μg/mL (MIC)	Echinomycin 0.0125 μg/mL (MIC)	[62]
	-	Serial agar diffusion/ *S. aureus* Smith	6.3 μg/mL (MIC)	Echinomycin 0.0125 μg/mL (MIC)	[62]
-	-	Serial agar diffusion/*S. pyogenes* S-23	0.8 μg/mL (MIC)	Echinomycin 0.0063 μg/mL (MIC)	[62]
-	-	Serial agar diffusion/ *Sarcina lutea* PCI 1001	6.3 μg/mL (MIC)	Echinomycin 0.0063 μg/mL (MIC)	[62]
-	-	Serial agar diffusion/ *Micrococcus flavus* D 12	6.3 μg/mL (MIC)	Echinomycin 0.0063 μg/mL (MIC)	[62]
-	-	Serial agar diffusion/ *Corynebacterium Xerosis* 53 K-1	6.3 μg/mL (MIC)	Echinomycin 0.2 μg/mL (MIC)	[62]
-	-	Serial agar diffusion/*B. subtilis* PCI 219	6.3 μg/mL (MIC)	Echinomycin 0.0031 μg/mL (MIC)	[62]
-	-	Serial agar diffusion/*B. megaterium* D2	1.6 μg/mL (MIC)	Echinomycin 0.1 μg/mL (MIC)	[62]
-	-	Serial agar diffusion/*B. anthracis* A 9504	1.6 μg/mL (MIC)	Echinomycin 0.025 μg/mL (MIC)	[62]
-	-	Serial agar diffusion/ *Mycobacterium smegmatis* 607 D 87	0.8 μg/mL (MIC)	Echinomycin 6.3 μg/mL (MIC)	[62]
-	-	Serial agar diffusion/ *Mycobacterium phlei* D 88	0.4 μg/mL (MIC)	Echinomycin 6.2 μg/mL (MIC)	[62]
-	Antiviral	HIV-1	0.4 μM (IC_50_)	Sandramycin 2.0 μM (IC_50_)	[62]
Quinoxapeptin A (63)	Antiviral	HIV-1	0.6 μM (IC_50_)	Sandramycin 2.0 μM (IC_50_)	[65]
-	-	HIV-1	4.0 nM (IC_50_)	-	[68]
-	-	HIV-2	40.0 nM (IC_50_)	-	[68]
-	Cytotoxicity	MTT/L1210	0.3 nM (IC_50_)	Sandramycin 0.001 nM (IC_50_)	[65]
-	-	MTT/HCT-116	1.0 nM (IC_50_)	Sandramycin 0.007 nM (IC_50_)	[65]
Quinoxapeptin B (64)	Antiviral	HIV-1	0.9 μM (IC_50_)	Sandramycin 2.0 μM (IC_50_)	[65]
-	-	HIV-1	10.0 nM (IC_50_)	-	[68]
-	-	HIV-2	100.0 nM (IC_50_)	-	[68]
-	Cytotoxicity	MTT/L1210	2.0 NM (IC_50_)	Sandramycin 0.001 nM (IC_50_)	[65]
-	-	MTT/HCT-116	7.0 nM (IC_50_)	Sandramycin 0.007 nM (IC_50_)	[65]
Quinoxapeptin C (65)	Antiviral	HIV-1	0.3 μM (IC_50_)	Sandramycin 2.0 μM (IC_50_)	[65]
Korkormicin A (66)	Antibacterial	Serial broth dilution/ Enterococcus faecalis A20688	0.5 μg/mL (MIC)	-	[69]
-	-	Serial broth dilution/*E. faecalis* A25707 (ATCC 29212)	0.5 μg/mL (MIC)	-	[69]
-	-	Serial broth dilution/*E. faecalis* A25708 (ATCC 33186)	0.5 μg/mL (MIC)	-	[69]
-	-	Serial broth dilution/ *Staphylococcus aureus* A9537	0.13 μg/mL (MIC)	-	[69]
-	-	Serial broth dilution/*S. aureus* A20698	0.25 μg/mL (MIC)	-	[69]
-	-	Serial broth dilution/*S. aureus* A24407	0.13 μg/mL (MIC)	-	[69]

## LIST OF ABBREVIATIONS

5637: The Human Bladder Carcinoma Cell Line
A549: Human Lung Adenocarcinoma Epithelial Cell Line
AGS: TRAIL-Resistant Human Gastric Adenocarcinoma
B16: Highly Metastatic Mouse Melanoma Cell Line
BC: Human Epidermal Carcinoma Of The Nasopharynx Cell Line
CH2Cl2: Dichloromethane
CH3CN: Acetonitrile
CHCl3: Chloroform
CNS: Central Nervous System
COP 1: Copolymer 1
DNMT: DNA methyltransferase
EC50: Half Maximal Effective Concentration
ECD: Electronic Circular Dichroism
EtOAc: Ethyl Acetate
FMCA: Fluorometric Microculture Cytotoxicity Assay
GABA: Gamma-Aminobutyric Acid
H2O: Water
HCT-116: Human Colon Cancer Cell Line
HDAC: Histone Deacetylase
HeLa: Human Cervical Epitheloid Carcinoma Cell Line
HepG2: Human Hepatocellular Liver Carcinoma Cell Line
HPLC: High-Performance Liquid Chromatography
HRESIMS: High Resolution Electrospray Ionization Mass Spectroscopy
IC_50_: Half-Maximal Inhibitory Concentration
ip: Intraperitoneally
IR: Infrared
K562: Human Erythroleukemic Cell Line
KB: Human Oral Epidermoid Carcinoma Cell Line
L1210: Mouse Lymphocytic Leukemia Cell Line
L5178Y: Mouse Lymphoma Cell Line
LD50: Half Maximal Lethal Concentration
LEWIS: Murine Lewis Lung Carcinoma
M 109: Human Lung Carcinoma Cell Line
MABA: Microplate Alamar Blue assay
MCF-7: Human Breast Adenocarcinoma Cell Line
MeOH: Methanol
MFH: N-methyl-N-formylhydrazine
MH: N-methylhydrazine
MIC: Minimum Inhibitory Concentrations
MTT: 3-(4,5-Dimethylthiazol-2-yl)-2,5-diphenyltetrazolium bromide
NCI-H187: Human Small-Cell Lung Cancer
NCI-H23: Human Lung Cancer Cell Line
NCI-H446: Human Lung Cancer Cell Line
NCI-H460: Human Non-Small Cell Lung Cancer Cell Line
NGF: Nerve Growth Factor
NMR: Nuclear Magnetic Resonance
P 388: Human Leukemia Cell Line
PANC-1: Human Pancreas Ductal Carcinoma Cell Line
RP-18: Reversed Phase-18
RT: Reverse Transcriptase
SF268: Human Astrocytoma Cell Line
SiO2 CC: Silica Gel Column Chromatography
SRB: Sulforhodamine B
T24: Human Urinary Bladder Cancer Cell Line
TLC: Thin layer chromatography
TRAIL: Tumor Necrosis Factor-Related Apoptosis-Inducing Ligand.
U251: Glioblastoma Cell Line
U87MG: Glioblastoma Cell Line
Vero cell: Normal African Green Monkey Kidney Fibroblasts
